# ﻿*Yersinochloanianheana* (Poaceae, Bambusoideae), a new species from southern Vietnam

**DOI:** 10.3897/phytokeys.247.132975

**Published:** 2024-10-08

**Authors:** Nong Van Duy, Tran Thai Vinh, Vu Kim Cong, Quach Van Hoi, Dang Thi Tham, Nguyen Thi Thanh Hang, Dinh Van Khiem, Hoang Thanh Truong, Nguyen Hoang Nghia, Nguyen Thi Ai Minh, Tran Van Tien

**Affiliations:** 1 Taynguyen Institute for Scientific Research, Vietnam Academy of Science and Technology, Dalat, Vietnam Taynguyen Institute for Scientific Research, Vietnam Academy of Science and Technology Dalat Vietnam; 2 Graduate University of Science and Technology, Vietnam Academy of Science and Technology, Dalat, Vietnam Graduate University of Science and Technology Dalat Vietnam; 3 Vietnam Forest Science Institute of Central Highlands and South of Central Vietnam, Vietnam Academy of Forest Science, Da Lat City, Lam Dong Province, Vietnam Vietnam Forest Science Institute of Central Highlands and South of Central Vietnam, Vietnam Academy of Forest Science Da Lat City Vietnam; 4 Vietnam Academy of Forest Science, Hanoi, Vietnam Vietnam Academy of Forest Science Hanoi Vietnam; 5 Dalat University, Da Lat City, Lam Dong Province, Vietnam Dalat University Da Lat City Vietnam

**Keywords:** Bambusoideae, morphology, new taxon, pseudo-spikelet

## Abstract

*Yersinochloanianheana***sp. nov.** from Vietnam is described and illustrated. It is found in southern Vietnam, where it occurs at an elevation of 1400–1500 m in Voi Mountain, Duc Trong District, Lam Dong Province. This new species is distinguished from a similar species, *Yersinochloadalatensis*, by culm nodes with a thick swollen patella, culm leaf blades erect, auricles conspicuous, margins bearing long hairs, palea dorsal view showing rachilla extension and rudimentary floret at the apex and lodicules purple gradually towards the top. Besides that, the species is distinguished from *Yersinochloanghiana* by the leaf blades without being swollen at the base, the prophyllate bud 2-keeled, lodicules purple at ½ upper parts and unbifid at the base.

## ﻿Introduction

*Yersinochloa* H.N.Nguyen & V.T.Tran is a genus that was established by Hoang Nghia Nguyen and Van Tien Tran in 2016, based on *Yersinochloadalatensis* H.N.Nguyen & V.T.Tran. It belongs to the subtribe *Bambusinae* J.S.Presl of the tribe Bambuseae (Poaceae, Bambusoideae) (Soreng et al. 2017). The genus consisting of two species are *Yersinochloadalatensis* H.N.Nguyen & V.T.Tran and *Yersinochloanghiana* V.T.Tran & T.V.Tran and is narrowly distributed in a degraded natural forest, in the south highlands of Vietnam (Nguyen and Tran 2016; Tran et al. 2023). *Yersinochloa* is distinguished from the other related genera of the subtribe *Bambusinae* by pseudo-spikelets with only one perfect floret, palea unkeeled and anther apices with tiny spines (Nguyen and Tran 2016).

During a bamboo survey in Voi Mountain, Duc Trong District, Lam Dong Province, southern Vietnam, in December 2023, the authors found several sparsely growing populations of climbing bamboo in a degraded natural forest of valleys, between 1400 and 1500 m a.s.l. Plants from these populations have only one perfect floret with no terminal vestigial flowers and the palea was unkeeled; the anther apex bore tiny spines. The form and structure of branches and inflorescences in the collected specimens are similar to *Yersinochloa*. However, the character states considered important at the species level for distinguishing clambering bamboo species are given in Table 1. As this combination of culm-leaf and inflorescence structure, along with the other features described here, is not found in any other bamboo species, we describe a new species from Vietnam, *Yersinochloanianheana*.

**Table 1.** Morphological comparisons of *Yersinochloanianheana* V.T.Tran, N.V.Duy. & T.V.Tran, sp. nov. with *Y.dalatensis* H.N.Nguyen & V.T.Tran and *Y.nghiana* V.T.Tran & T.V.Tran.

Characters		*Y.nianheana*	*Y.dalatensis*	*Y.nghiana*
Internode		culm nodes with a thick swollen patella	culm nodes without a thick swollen patella	culm nodes with a thick swollen patella
Culm leaves	culm-leaves blade	erect, tardily deciduous, uninflated at the base	Reflexed	erect, tardily deciduous, swollen at the base
	auricles	conspicuous, dense bristles	absent or inconspicuous	conspicuous, entire
Rachilla		0.1 cm	0.1 cm	0.5 cm
Palea		acute at apex	bifid at apex	acute at apex
Lodicules		lanceolate, purple gradually towards the top, acute at apex	obovate, purple, acute at apex	obovate or oblong, purple, bifid at base

## ﻿Materials and methods

This study was based on plant material collected from Voi Mountain, Duc Trong District, Lam Dong Province, southern Vietnam. The plant specimens were deposited at VTN-Taynguyen Institute for Scientific Research, DLU and VNMN-Vietnam National Museum of Nature. Vegetative parts were measured in the field; fresh flowers were examined under a Meiji Techno EM-32 stereomicroscope and colour photographs were taken using a camera Canon 600D. Other similar species were used for critical comparison.

## ﻿Taxonomic treatment

### ﻿ Yersinochloa nianheana

Taxon classificationPlantaePoalesPoaceae

V.T.Tran, N.V.Duy & T.V.Tran sp. nov.

D05474D6-A38A-58BC-B80F-6112D9698B02

urn:lsid:ipni.org:names:77349808-1

Figs 1 , 2

#### Diagnosis.

*Yersinochloanianheana* is morphologically most similar to *Y.dalatensis* and *Y.nghiana*, but *Y.nianheana* is distinguished from *Y.dalatensis* by culm nodes with a thick swollen patella (vs. without a thick swollen patella), culm-leaf blade erect (vs. reflexed) and auricles conspicuous (vs. absent). It also differs from *Y.nghiana* in culm-leaf blade flat at the base (vs. swollen at the base), palea with white cilia at the top (vs. glabrous), lodicules purple at ½ upper parts, unbifid at the base (vs. purple, bifid at base) and prophyllate bud 2-keeled (vs. prophyllate bud 1-keeled) (Fig. 3).

**Figure 1.**
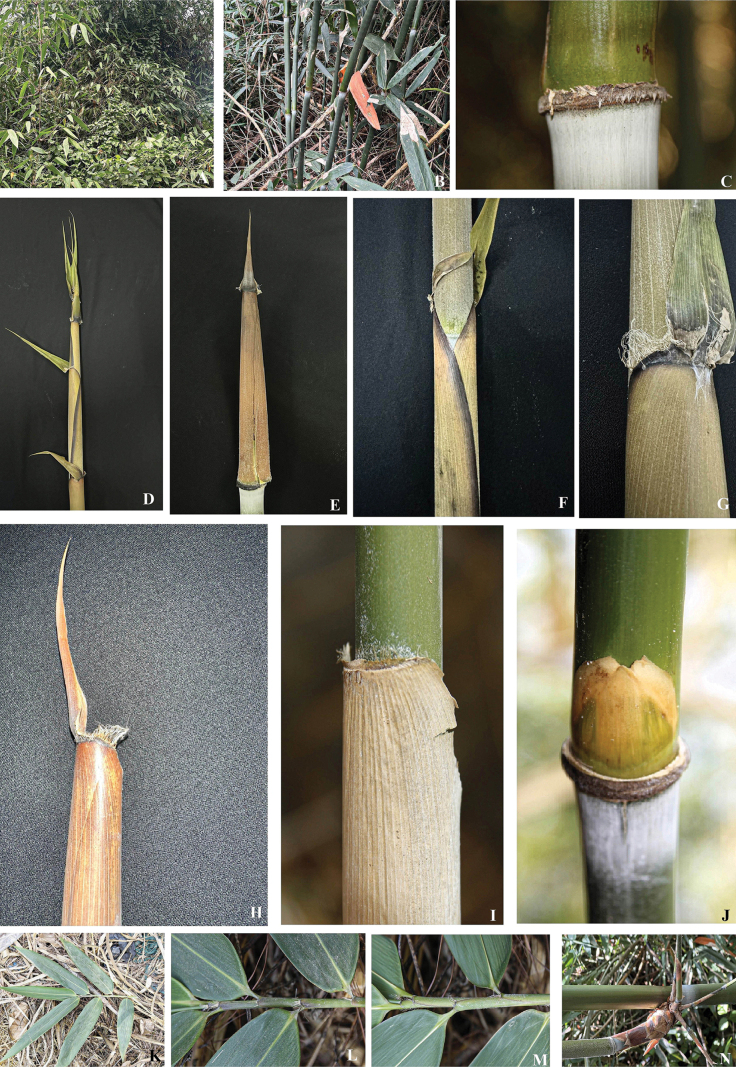
*Yersinochloanianheana* V.T.Tran, N.V.Duy & T.V.Tran **A** habitat **B** clump **C** node **D** shoots **E, F** culm leaves **G** auricles **H** culm leaf **I** ligule **J** bud **K** leafy branch **L, M** section of a leafy branch **N** several branches with middle one dominant. Photos by Tran Thai Vinh from the type locality.

**Figure 2.**
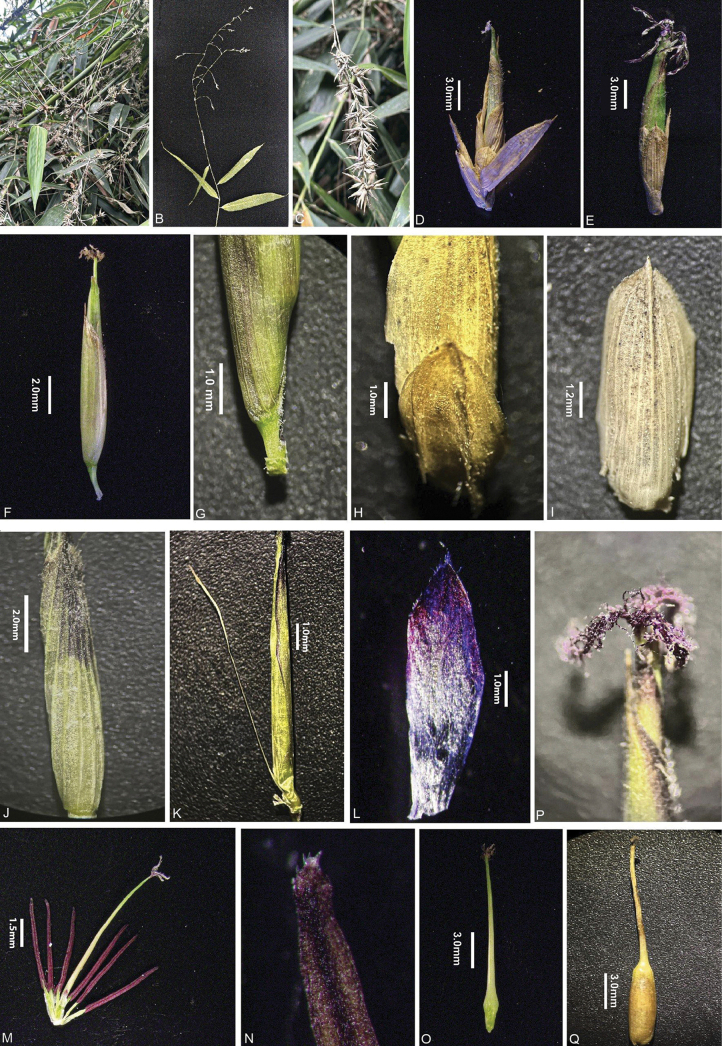
*Yersinochloanianheana* V.T.Tran, N.V.Duy & T.V.Tran **A, B, C** inflorescence terminating at leafy branches **D, E** pseudo-spikelets **F** perfect florets **G** rachilla internode **H** prophyllate bud 2-keeled **I** glume **J** lemma **K** palea with rachilla extension **L** lodicules **M** stamens and pistil **N** anther apiece bearing tiny spines **O** young fruit **P** stigmas **Q** mature fruit. Photos by Tran Thai Vinh from the type locality.

**Figure 3.**
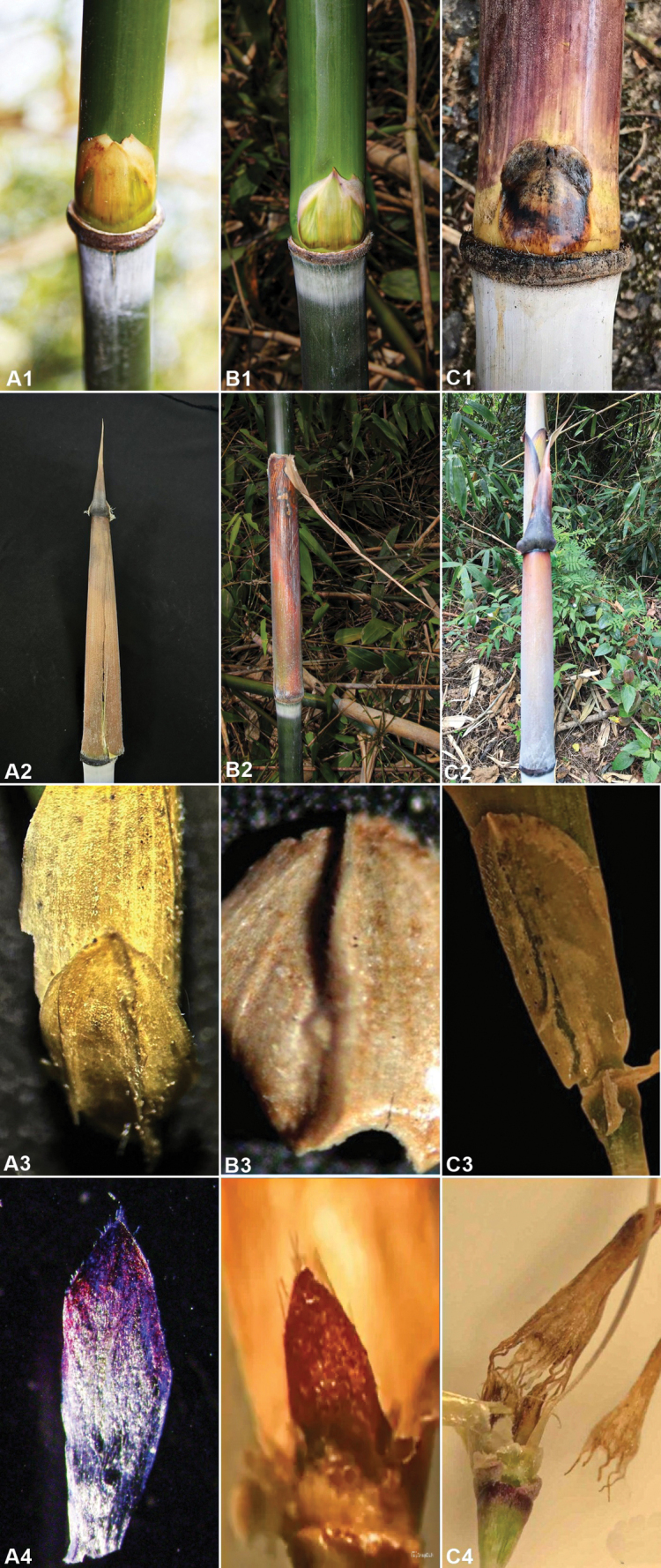
*Yersinochloanianheana* V.T.Tran, N.V.Duy & T.V.Tran, *Y.dalatensis* H.N.Nguyen & V.T.Tran and *Yersinochloanghiana* V.T.Tran & T.V.Tran **A1** bud **A2** culm sheath **A3** prophyll **A4** lodicules **B1** bud **B2** culm sheath **B3** prophyll **B4** lodicules **C1** bud **C2** culm sheath **C3** prophyll **C4** lodicules. Photos by Tran Thai Vinh from the type locality.

#### Type.

Vietnam • Lam Dong Province, Duc Trong District, Voi Mountain, 1420 m a.s.l., 11°48'21.02"N, 108°24'46.12"E, 20 Sep 2023, *N.V. Duy, V.T. Tran VTN 1990* (holotype DLU!; isotype VNMN!).

#### Description.

Culms and branches scrambling or hanging over nearby vegetation or trees, 5–8 m tall; internodes 50–70 cm long and 1.3–1.5 cm in diameter; white at the node when young, densely covered with adpressed white hairs; culm walls 0.4–0.5 mm thick; nodes with a thick swollen patella, hairy. Branches 5-7 with the middle one dominant and elongated. Culm leaves leathery purplish, sheaths with densely adpressed white hairs on the abaxial side; 25–27 cm long and 9–10 cm wide at the base, apex 6–7 cm wide; margins bearing dense white-brown hairs; blades cordate, slightly constriction at the base, erect, early deciduous, purple-brown, 14–18 × 3–4 cm, abaxial surface with dense white hairs at the base; auricles conspicuous, 1.0–1.2 × 0.1–0.2 cm; margins bearing dense white bristles, ca. 0.8–1.0 cm long; ligule short, ca. 1 mm, entire. Leafy branches bearing 8–10 leaves, foliage leaf blades wedge-shaped, 35–38 × 6–8 cm, acute or cuneate-obovoid at base, glabrous; veins 11–13 pairs; sheaths with ciliate margins, auricles with dense bristles 4–6 mm long; inner ligule with a low rim, ca. 1 mm; pseudo-petiole ca. 7–9 mm length, arching over. Inflorescences terminating at leafy branches, indeterminate; pseudo-spikelets typically 1.8–2.2 cm long, each subtended by a prophyllate bud, 2-keeled, with ciliate margins and hairy on 2-keels; 0.8–1.0 × 1.0–1.2 mm, apex acute, mucronate, ca. 0.5 mm long and consisting of one glume, one perfect floret. Rachilla internode below fertile floret ca. 0.1 cm. Fertile floret 1.0–1.2 × 0.2–0.4 cm; lemma oblong-lanceolate, 0.8–1.0 × 0.2–0.4 cm, veins 9–10, apex acute with 0.1 mm long, margins bearing dense white cilia; palea unkeeled, dorsal view showing rachilla extension and a rudimentary floret at apex, 1.0–1.2 × 0.5–0.6 cm, with margins bearing dense white cilia at the top, acute at apex, base inrolled; lodicules 3, lanceolate, purple gradually towards the top, ca. 0.3–0.4 × 0.1–0.2 mm, acute at apex, top with hairs 0.5 mm long, ciliate margins at ½ upper parts. Stamens 6; filaments free, 0.7–0.8 cm; anther ca. 5 mm, purple, apices bearing tiny spines, ca. 0.5 mm. Ovary green, glabrous with a long style, style oblique 1.0–1.2 cm; stigmas 3, purple; caryopsis oblique, with a relatively thin pericarp, 0.6–0.7 × 0.1–0.2 cm, with a long style, ca. 0.9–1.1 cm.

#### Distribution and habitat.

*Yersinochloanianheana* grows in degraded natural forest in the valleys, between 1400 and 1500 m a.s.l., in Voi Mountain, Duc Trong District, Lam Dong Province.

#### Phenology.

The plants were found flowering in December 2023. New shoots from June to August.

#### Local uses.

*Yersinochloanianheana* is of considerable importance to the local people. Its culms are used for making handicrafts and household tools.

#### Etymology.

The new species is named in honour of Prof. Xia Nianhe, for his contributions to bamboo research.

#### Preliminary conservation status.

*Yersinochloanianheana* is only known from a single population in Voi Mountain, Duc Trong District, Lam Dong Province, Vietnam. This population has no more than 500 mature clumps, all growing in degraded natural forests in valleys. According to IUCN Red List Categories and Criteria (IUCN 2022), the species is classified as data deficient (DD) and needs more surveys.

### ﻿Key to the species of *Yersinochloa* in Vietnam

**Table:** 

1	Leaf blade reflexed	***Y.dalatensis***
–	Leaf blade erect	**2**
2	Leaf blade swollen at the base	***Y.nghiana***
–	Leaf blade flat at the base	***Y.nianheana***

## Supplementary Material

XML Treatment for Yersinochloa nianheana

## ﻿Additional information

### Author ORCIDs

Nong Van Duy https://orcid.org/0000-0002-1509-4412

Tran Thai Vinh https://orcid.org/0000-0001-6410-1423

Vu Kim Cong https://orcid.org/0009-0005-4979-0802

Quach Van Hoi https://orcid.org/0000-0002-3691-941X

Dang Thi Tham https://orcid.org/0009-0008-8240-0126

Nguyen Thi Thanh Hang https://orcid.org/0009-0007-3534-8874

Dinh Van Khiem https://orcid.org/0009-0008-4398-0762

Hoang Thanh Truong https://orcid.org/0000-0002-8799-5818

Nguyen Thi Ai Minh https://orcid.org/0009-0000-7521-2065

Tran Van Tien https://orcid.org/0000-0001-6539-0327

### Data availability

All of the data that support the findings of this study are available in the main text.
